# Fighting Cancer with Photodynamic Therapy and Nanotechnologies: Current Challenges and Future Directions

**DOI:** 10.3390/ijms26072969

**Published:** 2025-03-25

**Authors:** Laura Marinela Ailioaie, Constantin Ailioaie, Gerhard Litscher

**Affiliations:** 1 Department of Medical Physics, Alexandru Ioan Cuza University, 11 Carol I Boulevard, 700506 Iasi, Romania; lauraailioaie@yahoo.com (L.M.A.); laserail_mail@yahoo.com (C.A.); 2 Swiss University of Traditional Chinese Medicine, SWISS TCM UNI, High-Tech Acupuncture and Digital Chinese Medicine, 5330 Bad Zurzach, Switzerland; 3 President of the International Society for Medical Laser Applications (ISLA Transcontinental), German Vice President of the German-Chinese Research Foundation (DCFG) for TCM, Honorary President of the European Federation of Acupuncture and Moxibustion Societies, Honorary Professor of China Beijing International Acupuncture Training Center, China Academy of Chinese Medical Sciences, Honorary President of the American Association of Laser Acupuncture Therapy (ASLAT), USA, Former Head of Two Research Units and the TCM Research Center at the Medical University of Graz, 8053 Graz, Austria

**Keywords:** cancer treatment, mitochondria, multifunctional theranostics platforms, nanoparticles, nanoscale delivery vehicles, nanoshuttles, neoplasms, PDT, photosensitizers, tumor hypoxia, TME

## Abstract

Photodynamic therapy (PDT) is an innovative treatment that has recently been approved for clinical use and holds promise for cancer patients. It offers several benefits, such as low systemic toxicity, minimal invasiveness, and the ability to stimulate antitumor immune responses. For certain types of cancer, it has shown positive results with few side effects. However, PDT still faces some challenges, including limited light penetration into deeper tumor tissues, uneven distribution of the photosensitizer (PS) that can also affect healthy cells, and the difficulties posed by the hypoxic tumor microenvironment (TME). In hypoxic conditions, PDT’s effectiveness is reduced due to insufficient production of reactive oxygen species, which limits tumor destruction and can lead to relapse. This review highlights recent advances in photosensitizers and nanotechnologies that are being developed to improve PDT. It focuses on multifunctional nanoplatforms and nanoshuttles that have shown promise in preclinical studies, especially for treating solid tumors. One of the key areas of focus is the development of PSs that specifically target mitochondria to treat deep-seated malignant tumors. New mitochondria-targeting nano-PSs are designed with better water solubility and extended wavelength ranges, allowing them to target tumors more effectively, even in challenging, hypoxic environments. These advancements in PDT are opening new doors for cancer treatment, especially when combined with other therapeutic strategies. Moving forward, research should focus on optimizing PDT, creating more efficient drug delivery systems, and developing smarter PDT platforms. Ultimately, these efforts aim to make PDT a first-choice treatment option for cancer patients.

## 1. Introduction

Research in recent decades on knowledge related to the occurrence, early diagnosis, development, and complex treatment methods of neoplasia has had a rapid evolution, reduced mortality, and improved the living conditions for millions of patients. However, the work of researchers must continue to answer all the key questions in basic, translational, and clinical cancer research that have not yet been addressed.

### 1.1. Biological Characteristics of Neoplasms

Neoplasms have six biological features gained during evolution, which provide complexity through the maintenance of proliferative signaling, evasion in the face of suppressive factors, resistance to cell death, immortality through perpetual replication, development of new blood vessels, strong expansion, and distant proliferation, all due to genome instability, which triggers genetic diversity and precipitates the emergence of all these hallmarks and inflammation [1].

### 1.2. Current Cancer Statistics and Future Projections

According to the latest statistical data from the American Cancer Society, 2,001,140 new cases of cancer and 611,720 deaths caused by this serious disease have been estimated for the US by 2024 [2].

According to the World Health Organization (WHO) and the International Agency for Research on Cancer, global cancer incidence and mortality are projected to expand, and the cancer burden will increase by about 77% by 2050, putting even more pressure on health systems, individuals, and communities [3].

### 1.3. Challenges in Drug Development and Potential Solutions

The diversity of cancers, some of which are unique, is challenging and requires innovative approaches in oncology pharmaceutical research, development, and manufacturing.

Accelerating new anticancer therapies to benefit patients worldwide must overcome several barriers, the most significant of which are high costs, long development and testing periods, and potentially low success rates. An analysis of the ten most recent anticancer drugs produced by ten US pharmaceutical companies revealed a median time to market of 7.3 years (range: 5.8–15.2 years) and a median cost of $648.0 million (range: $157.3 million–$1.95 billion).

In summary, the drug discovery and development processes involve many years, many failures, a lot of uncertainty, and, additionally, regulations, production, and finding the right patients for testing; if it does not work the first time, it is necessary to learn from mistakes and try again [4,5,6,7]. A promising solution in scientific research and the pharmaceutical industry would be computer-assisted drug discovery, which offers immense advantages for overcoming these obstacles and especially for accelerating the processes. Various machine learning techniques can fundamentally change the stages of drug discovery, from virtual screening to predictive modeling of drug-target interactions.

### 1.4. Role of Artificial Intelligence (AI) and Quantum Computing in Oncology

The great hope is opened by quantum computing, which is still in its early stages, but which very soon could be applied to find optimal solutions regarding the complicated aspects of molecular simulations and optimization, impossible for classical computers [8].

The hope of addressing the complexity of cancer from the perspective of discovering new drugs and innovative nanotechnologies also involves the use of AI algorithms to process the huge data generated by the evolution of cancer, from a single cell to metastases. The multifaceted nature of cancer as a systemic disease encompasses tumor emergence and development, tumor and immunological microenvironment, senescence, metabolic and nutritional disorders, circadian rhythms, involvement of the nervous system, microbiome and coagulation disorders. AI models of genetic mutations are fundamental for analyzing and interpreting gene-environment interactions, providing molecular oncology with valuable information repositories for cancer prevention, early diagnosis, improvement of quality of life, and provision of targeted therapy. AI provides a diverse palette of computational tools and algorithms, which facilitate the speeding up of drug discovery processes, making the best of nanoformulation development, upgrading manufacturing efficiency, strict quality control and revolutionizing post-market surveillance methodologies [9,10,11].

### 1.5. Evolution of Cancer Therapies

The historical axis of anticancer therapies began long ago, and some are still administered today. In the second half of the 20th century, the initiation of cytotoxic antitumor therapy in solid and hematological neoplasms through applied chemotherapy constituted a cornerstone in cancer treatment. Advances in the 1980s, as a result of research in cellular and molecular biology, led to the synthesis of molecularly targeted cancer drugs. Innovative studies related to oncogenic genes and the signaling pathways involved have represented a major step forward in cancer treatment. Chemotherapy and targeted therapy have substantially improved the quality of life of patients, survival and the complete disappearance of some tumors. However, the inability to target some key oncogenic mutations, the acquisition of drug resistance after favorable preliminary clinical results, toxicity and other subsequent side effects have represented major barriers.

At the beginning of the 21st century, genetic engineering triggered a new stage in clinical pharmaco-oncology therapy by introducing monoclonal antibodies and immune checkpoint inhibitors for serious or even metastatic neoplasms, which previously offered no hope.

Currently, new cell therapies, antitumor vaccines and innovative biopharmaceutical products have emerged with great hopes for the future [12,13,14].

The up-to-date surgical, radiological and pharmacological treatments used in oncology, although they have reduced the heavy burden of cancer worldwide, have not achieved complete eradication, and have not stopped some relapses, toxic side effects and immune system disorders.

### 1.6. Innovations in Photodynamic Therapy (PDT)

Current integrative multimodal therapies, such as PDT, photothermal therapy (PTT), photoimmunotherapy, and smart multifunctional platforms loaded with nanodrugs, are innovative solutions in nanomedicine and hold promise for synergistic cancer treatment in the future [15]. PDT, as a cancer treatment method, involves selective sensitization of tissues to light, and has opened the era of nanotechnology [16].

**Figure 1:**
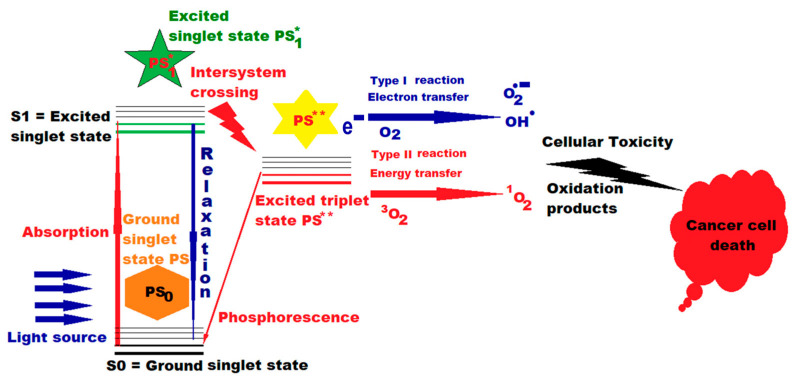
Action of PDT and photosensitizer (PS) in cancer therapy. A single asterisk * means “excited singlet state”, and two asterisks ** mean “excited triplet state” (Figure 1 was imagined and drawn by L.M.A. using Microsoft Paint 3D for Windows 10, being already published by L.M.A. [21]).

PDT is minimally invasive, does not induce significant adverse reactions, and triggers antitumor immune responses. Although preclinical and clinical results of PDT have been favorable, and it has received U.S. Food and Drug Administration (FDA) approval since 1995 for the treatment of esophageal cancer, it is not yet widely applied in clinics worldwide due to some limitations. These impediments are the reduced penetration of light into the depth of the tumor mass; the biodistribution of photosensitizer (PS) that can also accumulate in healthy cells; insufficient number of reactive oxygen species (ROS) due to hypoxic tumor microenvironment (TME); transient vascular injury and then repair; incomplete tumor destruction, with sub-sequent tumor recurrence; and limited generation of antitumor immune responses. For PSs to become ideal, it is necessary to improve their solubility, photostability, selectivity and phototoxicity by applying the latest nanotechnologies. PDT still needs to be improved by assimilating new nanotechnologies and overcoming all the current limitations mentioned above, to become a first-choice treatment for patients. Combining PDT with other therapies, performed through diverse strategies, can generate cooperative or synergistic effects to increase efficiency and achieve the eradication of the tumor [17,18,19,20] (Figure 1).

### 1.7. Future Directions in Cancer Research

Research in recent years has shown that some photosensitizers incorporated into nanotechnologies have demonstrated higher intracellular uptake, superior targeting rates, increased toxicity to cancer cells, accelerated caspase activity, and deoxyribonucleic acid (DNA) cleavage, ultimately triggering apoptosis [21]. PDT as a state-of-the-art medical technology, using blue light (400–470 nm), can also effectively fight bacterial, fungal, and viral infections [22].

Innovative strategies for future research should include the development of new radicals, the design and manufacturing of new light-activatable PSs in the TME, the disruption of the electron transport chain in the mitochondrial membrane by photocatalytic oxidation of NADH in complex I, and the innovation of smart PDT systems [23].

**Figure 2:**
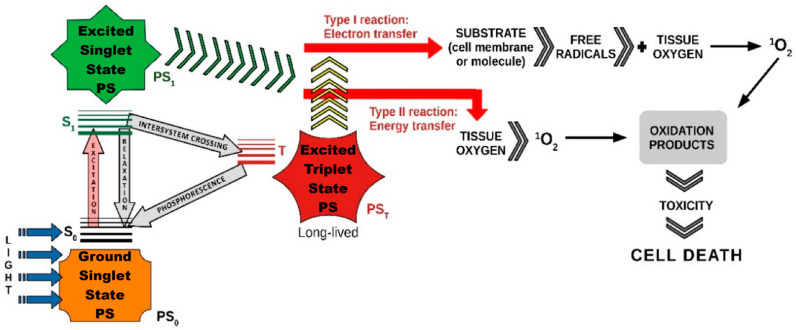
Comprehensive action of PDT in malignant tumors (Figure 2 was imagined and drawn by L.M.A. using Microsoft Paint 3D for Windows 10, being modified after Figure 3 [21]).

**Figure 3:**
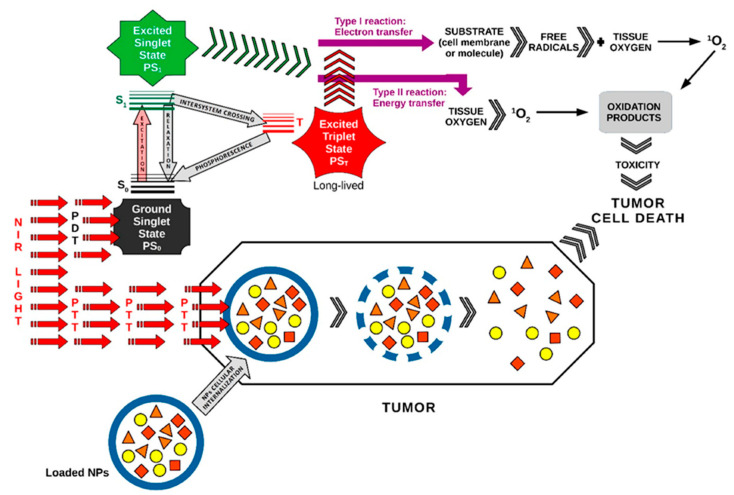
PDT and PTT applied synergistically in cancer therapy. {Figure 3 was imagined and drawn by L.M.A. using Microsoft Paint 3D for Windows 10, being modified after Figure 1 [15]}.

Various therapeutic modalities, including PTT, PDT, X-ray induced PDT (XPDT), immunotherapy, and synergistic therapy, have been extensively explored for effective disease treatment. Each of these approaches offers unique advantages: PTT relies on localized heat generation to destroy diseased cells, XPDT enhances PDT’s applicability by enabling deeper tissue penetration, and immunotherapy strengthens the body’s immune response against pathological conditions. Synergistic therapy, which combines multiple treatment strategies, has emerged as a promising approach to enhance therapeutic outcomes. Among these, PDT has gained particular attention due to its minimally invasive nature, precise tumor targeting, and ability to induce localized cytotoxic effects with minimal damage to surrounding healthy tissues. Furthermore, its compatibility with other therapeutic strategies highlights its crucial role in advancing more effective and personalized treatment approaches (Figure 2 and Figure 3).

The primary aim of this review was to present a synthesis of current photosensitizers and the nanotechnologies in which they can be incorporated as PDT nanoagents, multifunctional theranostic nanoplatforms, and nanoshuttles successfully applied in preclinical studies of photodynamic therapy, with a focus on solid tumors.

The secondary goal was to highlight studies on PDT applications mainly targeting mitochondria for the elimination of deep malignant tumors.

Starting from the studies reviewed, the final objective is to invite and encourage researchers to contribute through creative approaches to optimizing PDT performance, imagining and designing new high-efficiency platforms for pharmaceutical formulations, smart PDT systems, and nanoshuttles through interdisciplinary approaches, also involving AI tools and computational algorithms to accelerate the discovery processes of innovative bioformulations for cutting-edge applications in molecular oncology and clinical cancer therapy.

## 2. State-of-the-Art Nano-Photosensitizers, Functional Nanosystems and Nanoscale Delivery Vehicles

Accurate image-guided treatment is of paramount importance in eliminating tumors in clinical applications. Mao et al. investigated an innovative nanotechnology using a chemiexcited photosensitizer, which can be precisely stimulated by hydrogen peroxide (H_2_O_2_) within the neoplastic surroundings to produce far-red/near-infrared (FR/NIR) emission and singlet oxygen (^1^O_2_). Organic fluorogens with aggregation-induced emission (AIEgen) features have emerged as favored materials for nanoparticle (NP) manufacture. AIEgens exhibit low fluorescence in solutions, but this phenomenon becomes stronger in aggregates due to limited intramolecular motion. Bis [2,4,5-trichloro-6-(pentyloxycarbonyl)phenyl] oxalate (CPPO) and an AIE photosensitizer, TPE-BT-DC (TBD), were carefully designed for aggregation-induced FR/NIR emission and co-encapsulated in pluronic F-127 and soybean oil to form C-TBD nanoparticles (C-TBD NPs). Using these NPs, breast tumors (both primary and metastatic) can be detected clearly through chemiluminescence imaging with a very high signal-to-noise ratio. These NPs serve as specific H_2_O_2_ probes for in vivo cancer detection through chemiluminescence imaging. The chemiluminescent signals from tumors and the ^1^O_2_ production can be enhanced by the addition of an antitumor drug, β-phenylethyl isothiocyanate (FEITC), which increases the amount of H_2_O_2_ at the tumor site for efficient neoplastic therapy. This demonstrates an advanced approach for light-source-free image-guided tumor treatment. C-TBD NPs open a new strategy for smart, accurate, and non-invasive tumor management [24].

### 2.1. Addressing Oxygen Deficiency in Tumor Environments

A deficiency in the amount of oxygen in nearly all solid tumors is a major limitation of conventional PDT and significantly reduces its curative success. Li et al. developed a molecular superoxide radical (O_2_–•) generator called ENBS-B, activated by NIR light, to overcome this challenge in hypoxic solid tumors. The authors revealed the O_2_–• mechanism responsible for antihypoxic reactions and validated its action by targeting hypoxic solid tumor ablation in vivo. This research demonstrates that near-infrared light-initiated molecular superoxide radical generators, paradoxically, under critical hypoxic conditions (only 2% O_2_), can produce significant O_2_–• through type I photoreactions. O_2_–• is partially converted to highly toxic OH· through SOD-mediated cascade processes. These radicals synergistically damage lysosomes inside cells, triggering apoptosis of cancer cells and resulting in potent PDT effectiveness [25].

### 2.2. Two-Photon Excited Fluorescence for Deeper Tumor Imaging

Sun et al. designed and tested organic dyes that exhibit excellent performance in two-photon excited fluorescence and ^1^O_2_ generation, such as turn-on-type two-photon absorption (2PA) dyes, which function even in intracellular environments and are suitable for both imaging and PDT of tumors in deeper tissues. They created an acetyl-terminated distyrylbenzene derivative (Ace-DSB), which quickly converts into aldehyde-terminated molecules (Ald-DSB), displaying a significant increase in 2PA cross-section (δ) values at 760 nm. This transformation activates the 2PA features of the molecules, enabling their use in intracellular conditions. Moreover, Ald-DSB showed a 40-fold higher capacity for 2P generation of ^1^O_2_ than Ace-DSB, paving the way for turning-on 2PA molecules for both in vitro and in vivo solid tumor imaging and 2PA-PDT. The turn-on feature was found useful for both two-photon laser confocal scanning microscopy imaging and two-photon excited PDT. The two techniques have been successfully applied to MCF-7 cells and melanoma tumors. This study sets the stage for the intelligent design of 2PA photosensitizers for future translational solid tumor treatments in clinical settings [26].

### 2.3. Covalent Organic Frameworks (COFs) for Photodynamic Therapy

A new category of organic porous materials, covalent organic frameworks (COFs), has shown promise in heterogeneous catalysis, energy storage, and analytical chemistry, with significant potential in cancer nanotherapeutics. COFs are characterized by porosity, modularity, stability, and metal-free structures, though size and structure control in aqueous dispersibility could improve their use. Guan et al. developed two boron-dipyrromethene (BODIPY)-decorated nanoscale COFs (NCOFs), which were nanocrystallized to be used for PDT of tumors. The two organic photosensitizers (PSs), BODIPY NCOFs, were obtained through Schiff-base precipitation of free end –CHO groups using the “functionalization of bonding defects” (BDF) method. These NCOFs had nanometric size (approximately 110 nm), low toxicity in the dark, and exceptionally high phototoxicity, proven by both in vitro and in vivo tests [27].

### 2.4. Multifunctional Nanoplatforms for Targeted Tumor Management

Neoplastic tumors are a significant challenge in medical science due to their variety, complexity, and the pressure to discover effective treatments. To address this, Wu et al. developed a multifunctional nanoplatform with intelligent tumor-specific theranostic capabilities for effective cancer management. A nanosponge composed of tightly packed MnO_2_-laden black phosphorus nanosheets (BPN) was designed as an ingenious, multifaceted theranostic nanocarrier. This platform enables tumor microenvironment (TME) simulation for pH and redox-responsive Magnetic Resonance Imaging (MRI) and synergistically enhances PDT, PTT, and chemotherapy. The (BPN/MnO_2_) nanoframework serves as a nanomedicine platform for tumor-specific smart diagnosis and autonomous treatment. The authors demonstrated that BPN/MnO_2_ PDT improves hypoxia by 3.8-fold and enhances PTT by 37% compared to BPNs alone. The MnO_2_ platform offers many TME-responsive properties, maximizing drug management at the neoplasm site and reducing chemotherapy side effects on healthy cells. An additional payload of doxorubicin (DOX) led to a BPN/MnO_2_/DOX nanocomposite, which exhibited smart self-controlled drug delivery under TME-specific functional reactions, such as pH, H_2_O_2_, and glutathione, with further control by external photoirradiation. This innovation demonstrates the potential of multifunctional nanotheranostics in cancer treatment [28].

### 2.5. Carbon Dots for Photodynamic Therapy

Carbon dots (CDs) have gained attention as nature-friendly nanomaterials with various applications. However, the development of CDs has faced constraints due to strict reaction conditions and lengthy synthesis steps. Wei et al. synthesized highly crystalline and luminescent CDs for the first time using a “wet chemistry” ion-thermal strategy, which allows the efficient production of CDs from different biochemical intermediates under non-severe conditions at ambient temperature and 210 °C. ZnCl_2_ was used to facilitate pyrolysis, developing CDs with tunable wavelengths and strong photogeneration of the superoxide radical anion (O_2_^−^) for high-tech applications such as PDT and photocatalysis, including the organic photosynthesis of tetrahydroquinoline via photosensitized cyclization [29].

### 2.6. Oxygen-Generating PDT Nanoagents

The ultimate effect of PDT in solid tumors relies on molecular oxygen in tissues, which must generate increased concentrations of cytotoxic ^1^O_2_, a process that is hindered by the hypoxia characteristic of solid neoplasms. Wang et al. developed a secure multifaceted self-assembled PDT nanoagent, namely OxgeMCC-r single-atom enzyme (SAE), consisting of a single-atom ruthenium as the active catalytic site attached in a metal–organic skeleton Mn_3_[Co(CN)_6_]_2_ with encapsulated chlorine e6 (Ce6), which serves as a cata-lase-like nanozyme for oxygen generation. Self-assembly of OxgeMCC-r SAEs requires in-group coordination, hydrophobic, and electrostatic interactions between organic linker, PVP polymer, photosensitizer, and metal ions. Setting up of Mn_3_[Co(CN)_6_]_2_ nanoparticles, referred to as MC, was achieved in a milky colloidal suspension, and these were nearly spherical, with a diameter of approximately 80 nm. Ru^3+^ was added to the initial solution and the doped Ru nodes were reduced with NaBH_4_. The newly created Mn_3_ [Co(CN)_6_]_2_-Ru (MC-r) nanoparticles also had a uniform size of approximately 80 nm. Ce6 was selected as the hydrophobic PS, to be integrated within MC or MC-r nanoparticles during the multicomponent self-assembly procedure. This operation gives rise to a nanometer scale matrix that is designed to encase Ce6. OxgeMCC-r SAE is characterized by precise form and structure, steady dimensions and big loading capability. Upon in situ production of O_2_ through the transformation between endogenous H_2_O_2_ and single-atom Ru species forming OxgeMCC-r SAE, the deficiency in the amount of oxygen in the tumor microenvironment is ameliorated. Wang et al.’s study provides a practical demonstration of a self-assembled nanozyme with truly organized and catalytically active single-atom Ru sites for PDT [30].

### 2.7. Lanthanide-Triplet Near-Infrared (NIR) Sensitization

Zheng et al. designed and tested an innovative procedure to reach high effects for ROS production via lanthanide-triplet NIR sensitization (for deeper penetration), in which lanthanide NP-organic PS nanoconjugates directly shifted the triplet energy from NPs to PSs, evading the intersystem crossing. This technique makes operational energy-effective NIR photosensitization to give rise to cytotoxic ^1^O_2_ for in vivo deep-tumor PDT under a low near-infrared power density of only 80 mW/cm^2^, i.e., 100 times smaller than conventional approaches. The researchers put together a NaGdF_4_:Nd (50 mol %) -Ce6 donor-acceptor hybrid system and they conducted systematic experiments to examine all of its photophysical properties. Ce6, as a standard PS, has been widely used in PDT studies for various cancer tumors and could be an energy acceptor with the lowest triplet state at 1.14 eV, making NIR photosensitization possible. The authors used an 8 nm NaGdF_4_ nanocrystal as a host medium in which they integrated Nd^3+^ as a photon absorber to make the Ce6 triplets sensitive. To demonstrate the influence of energy coupling on triplet relocation, they tested the process of inducing photosensitivity by lanthanide nanocrystals mixing different porphyrin and phthalocyanine derivatives (PpIX, TCPP, Ce6, and ZnPcS), characterized by certain triplet energies. They proved that only TCPP (T 1 = 1.43 eV9), Ce6 (T 1 = 1.14 eV), and ZnPcS (T 1 = 1.13 eV9) could be sensitized with maximum productivity by NaGdF_4_:Nd nanocrystals, when illuminated at 808 nm. Triplet levels of various porphyrin and phthalocyanine PSs could be successfully occupied by energy delivered from lanthanide NPs, without the necessity to populate the higher excited singlet states, so that the generation of cytotoxic ^1^O_2_ is feasible at ultralow NIR irradiation, opening new avenues for deep-tumor PDT [31].

### 2.8. Oxygen-Independent PDT via RNA-Targeting Photosensitizers

Being accurate and non-invasive, PDT is recognized as an elective treatment option for cancer. Even so, its conventional configuration relies on oxygen and ROS generation to eliminate the cancerous tumors based on type-I and type-II processes. At the same time, the sensitization of PSs, the medicines used against cancer cells, is based on the surrounding O_2_, which is insufficient in tumors, characterized by a significant lack of molecular oxygen, thus restricting the final curative outcome of PDT. Yao et al. invented a novel category of PSs, NBEX (X = S, Se, Te), which could attach to RNA of the neoplastic cells, and straightly induce the excitation energy to RNA for its breakdown after the irradiation by light, without the need for molecular oxygen, and thus destroying RNA and killing the corresponding neoplastic cells, even under hypoxic conditions, totally independent of O_2_, i.e., type III mechanisms. Previously, a smart fluorescent dye NBE was prepared, having its origins on the “door bolt” tool, which could identify RNA specifically and keep away from errors, recognizing cancer cells from normal ones due to the high level of RNA in neoplastic cells. The structure of NBEX was like that of NBE. In vivo, NBEX (X = S, Se, Te) could self-assemble into NPs and spontaneously gather into the tumor region within minutes, displaying an outstanding activity for eliminating various tumors and their metastases. At the same time, PDT with NBEX could influence and enhance immune responses to prevent further actions and relapses [32].

### 2.9. BODIPY-Based Photosensitizers for PDT

Among organic PSs, boron dipyrromethene (BODIPY) derivatives have captured the attention of researchers because they are fluorescent in the NIR with limited width of ab-sorption and emission bands, with very good fluorescence and photostability, increased elimination coefficient, and very good fluorescence quantum output. The implementations of BODIPY derivatives are multiple, both in the pharmaceutical field and in cancer therapy for targeted drug delivery, and as PSs, drug monitoring and tracking, as well as cancer diagnosis and treatment due to their fluorescence properties. Modifying the hydrophilic structure while maintaining the hydrophobic BODIPY as the core would improve the absorption range, would increase water solubility and biocompatibility for better use in living tissues. Various applications have been attempted, but all have proven expensive, difficult to achieve and manufacture, and with uncertain results by embedding hydrophilic protein-based formations, lipidosomes, and PEG substances into the hydrophobic BODIPY core [33,34,35,36].

Lu et al. [37] designed and prepared three new PSs as NIR BODIPY-CDs complexes (BODIPY-α-CD, BODIPY-β-CD, and BODIPY-γ-CD) with superior water solubility and biocompatibility through the click reaction of alkynyl-containing BODIPY and azide-modified cyclodextrin (CD). Because of stronger conjugation and after embodying triazole and CD elements, the fluorescence was displaced to the red region by approximately 90 nm. All three BODIPY derivatives had no cytotoxicity against NIH 3T3 and HeLa tumor cell lines under dark conditions. The BODIPY-β-CD type had the best PDT effect upon NIR irradiation (808 nm) against HeLa cells, with a drastic elimination of up to 80% neo-plastic cells, for a concentration of 100 μg/mL. BODIPY-CDs complexes offer new perspectives in PDT applied to cancer therapy [37].

### 2.10. Advances in PDT for Glioma and Deep Tumors

Glioma is a cancerous brain tumor and is challenging because its rate is 40% of all brain tumors. It is a virulent, intrusive cancer with a high recurrence and a likely poor course. All conventional means that could be applied, such as surgical operation, radio- and chemotherapy, are unsuccessful in eliminating this kind of tumor in 89% of patients, in relation to its position, the degree of penetration into the surrounding tissues and its size [38,39,40,41].

PDT has arisen as an encouraging novel anti-glioma treatment, but enhancements are needed by overcoming intra-tumoral hypoxia and extending the laser penetration into the neoplastic tissue [42,43].

Li et al. [44] designed and produced upconverted UCNPs@mSiO_2_-NH_2_ NPs by thermal breakdown, applying energy resonance transmission and NIR up-conversion. To study and control in time, space, and for accurate measurement of doses of the nitric oxide (NO) released in multiple biological implementations, the authors designed an 808 nm-excited NO photocontrolled release platform, by electrically charging at rest the photosensitive NO donor metal-nitrosyl complex, Roussin’s black salt (RBS), which releases NO after illumination onto UCNPs@mSiO_2_-NH_2_ NPs, resulting UCNPs@mSiO_2_-NH_2_&RBS with anti-neoplastic consequences [44].

NO is a vital signaling molecule, with major consequences in pathological activities, especially in cancer, where at high concentrations it generates reactive nitrogen species, which interreact with ROS and result in oxidative and nitrosyl reactions. These latter responses trigger DNA base deamination and enzyme nitrosylation, with important damage to cellular metabolism and, ultimately, cause programmed cell death of neoplastic cells [45,46,47].

In vivo investigations on glioma and chordoma xenograft mouse models demonstrated remarkable suppression of neoplastic cells in the UCNPs@mSiO_2_-NH_2_&RBS lot treated with NIR, without major adverse reactions in mice.

The UCNPs@mSiO_2_-NH_2_&RBS platform developed by the authors with NO release upon NIR irradiation has great prospects for PDT applied in deep malignant tumors [44].

Nanobiotechnology has advanced greatly and has paved the way for enzyme loading on structured nanomaterials with novel physical and chemical properties, related to the enhancement of catalytic quality and enzyme encapsulation [48]. To increase the potential of PDT, novel enzyme-encapsulated nanomaterials have been developed to target deep tumors with high-performance PS and trigger oxygen generation, which can rely on cata-lase-loaded nanodrugs or nanocarriers [49].

Qiao et al. designed and implemented a multifunctional PS consisting of catalase-loaded manganese-porphyrin frameworks (CAT@MnPFs) to catalytically aid PDT applied to 4T1 breast cancer cell line. The assembly of Mn^2+^ ions and Protoporphyrin IX (PpIX) occurred in a first step in MnPFs via covalent coordination, and then the catalase was loaded. Under irradiation with red light (wavelength = 650 nm), the porphyrin (protoporphyrin IX) in the CAT@MnPFs structure can transform oxygen (O_2_) into cytotoxic ^1^O_2_, which will trigger the destroying PDT effect. The packed catalase can break down the hydrogen peroxide (H_2_O_2_) into O_2_ in just 600 s, and, consequently, further advances the production of ^1^O_2_ through PDT, further enhancing the effects of photodynamic therapy.

Newly designed CAT@MnPFs cover the benefits of PSs and catalase for PDT characterized by evolving oxygen generation processes for the treatment of cells lacking oxygen in their environment, i.e., hypoxic media [50].

A synthesis of the studies presented above regarding some recent nanotechnologies tested in PDT is presented in Table 1.

## 3. Nano-Photosensitizers in PDT Targeting Mitochondria in Cancer Cells

### 3.1. Mechanisms of PDT and Challenges in Solid Tumors

Experimental studies conducted in vitro and in vivo, and clinical studies with different PSs have revealed the fate of target cells and their relationship with apoptosis, necrosis, autophagy, and other nonspecific aspects of cell death. Both the mode of action and the efficacy of PDT are facilitated by the release of ROS and correlate directly with the irradiation energy, dose, irradiation cycle, PS accumulation in the tumor, PS metabolism, and tumor hypoxia [51].

One of the limitations of PDT efficacy on solid tumors is hypoxia, which increases due to oxygen consumption during the therapeutic process. Consequently, overcoming this effect of PDT on tumor hypoxia remains a constant concern for researchers.

### 3.2. Advances in Nano-Photosensitizers for Hypoxia Mitigation

Yang et al. manufactured the Mn_3_O_4_@MSNs@IR780 nanoparticle by absorbing the PS IR780 into 90 nm mesoporous silica nanoparticles (MSNs) and coating the surface pores with 5 nm Mn_3_O_4_ nanoparticles. Under PDT, this nanoparticle exhibits a durable ability to reduce hypoxia through the catalysis of intratumoral H_2_O_2_ and mitochondrial damage targeted by IR780. This nanoparticle generated oxygen, ROS, and inhibited respiration in vitro on the human gastric cancer MKN-45P cell line and in vivo in MKN-45P tumor-bearing xenograft mice, preventing tumor recurrence and leading to a favorable prognosis [52].

Wen et al. [53] designed a nanoplatform with an oxygen regulator, 3-bromopyruvate (3BP), using a core/shell structured poly(lactic-co-glycolic acid) (PLGA) nanoplatform, with 3BP enclosed in the core and IR780 in the shell [3BP@PLGA-IR780], to enhance PDT.

**Figure 4:**
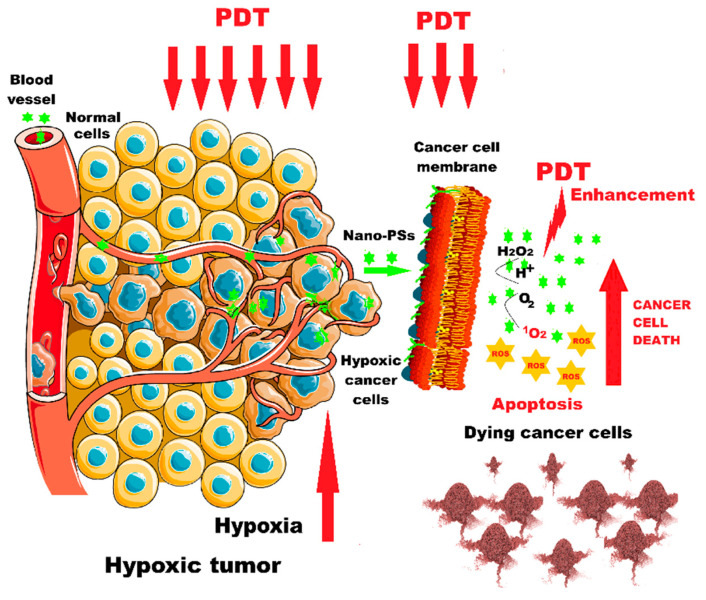
PDT enhancement in hypoxic tumors using nano-photosensitizers. Nano-PSs = nano-photosensitizers (Figure 4 was imagined and drawn by L.M.A. using Microsoft Paint 3D for Windows 10 and using completely free picture material from SeekPNG.com (accessed on 9 March 2025), for which we are very grateful).

For in vivo evaluation, 4T1-xenograft tumors were intravenously injected with the 3BP@PLGA-IR780 suspension. Experimental studies in 4T1-xenograft mammary tumor mice demonstrated that this nanoplatform has deep tumor penetration capabilities, disrupts tumor cell energy metabolism, reduces oxygen consumption by inhibiting the respiratory chain, enhances ROS generation, and precisely targets mitochondria. This platform could also function as a contrast agent for fluorescence and photoacoustic imaging in image-guided cancer treatment, making it a promising alternative for improving PDT performance [53] (Figure 4).

### 3.3. Novel Photosensitizer Strategies to Improve PDT

Zinc phthalocyanine (ZnPc) photosensitizers, despite their strong absorption at 650–750 nm, high triplet state quantum yields, and good biocompatibility, are rarely used in cancer therapy due to their low solubility and aggregation tendency in aqueous media. Nash et al. developed a novel nanoscale metal-organic layer (nMOL) assembly, ZnOPPc@nMOL, in which ZnOPPc (zinc(II)-2,3,9,10,16,17,23,24-octa(4-carboxyphenyl) phthalocyanine) PS was supported on secondary building units (SBU) of an Hf12 nMOL for PDT.

Upon laser application, SBU-bound ZnOPPc PSs absorbed 700 nm radiation and rapidly released singlet oxygen, preventing aggregation-induced self-quenching. ZnOPPc@nMOL exhibited mitochondrial penetration and tumor growth inhibition exceeding 99%, achieving cure rates of 40–60% in two colon cancer mouse models [54].

Given the remarkable photophysical properties of some PSs, they could be administered as multifunctional theranostic agents that combine PDT with deep penetration capabilities and high imagistics resolution. However, most PSs are not used due to low water solubility, poor specificity, and high toxicity in the dark. Cai et al. designed and encapsulated cyclometalated iridium (III) complexes (Ir) into biocompatible nanoparticles [Ir-NPs] that exhibited aggregation-induced emission (AIE) properties, high photoinduced ROS generation efficiency, two-photon excitation, and high mitochondrial targeting quality. The Ir-NPs disrupted mitochondrial redox homeostasis and in vitro apoptosis in SKOV3 ovarian carcinoma cell line. In vivo studies in mice bearing SKOV3 ovarian carcinoma tumors showed that Ir-NPs targeted, deeply penetrated, and destroyed the tumors with low toxicity in the dark. The authors reported that the Ir-NPs could be used as two-photon imaging agents for tissue visualization down to 300 μm. This multifunctional Ir-NPs nano-complex may serve to improve photodynamic therapy and clinical bioimaging applications [55].

### 3.4. Mitochondrial Targeting for Enhanced PDT in Breast Cancer

According to WHO statistics from 2022, breast cancer was diagnosed in 2.3 million women worldwide, leading to 670,000 deaths. Of all breast tumor subtypes, triple-negative breast cancer (TNBC) accounts for ~15% of invasive cases and is considered the most aggressive molecular subtype, exhibiting high metastatic potential, treatment resistance, and poor prognosis. While standard treatments include surgery, radiotherapy, chemotherapy, hormonal therapy, and targeted biologicals, major challenges persist due to toxicity, high recurrence rates, and therapeutic resistance [56,57,58].

Consequently, there is an urgent need to design and obtain new drugs or novel load-ing and delivery technologies that improve the clinical response of subjects with triple-negative breast cancer. PDT is an already approved method as an antitumor therapy, appreciated as a promising means of photoactivation, precise tumor targeting and low toxicity. However, due to the low water solubility of current PSs, rapid depletion of the optimal level of ROS due to high accumulations of glutathione (GSH) in TME, the efficacy of PDT and synergistic therapies is limited. Huang et al. designed and obtained the TPP-TK-PPa/DEM NPs nanoplatform which was prepared by self-assembly of the amphiphilic heterodimeric photosensitizer (TPP-TK-PPa) with 1,2-distearoyl-sn-glycero-3-phosphoethanolamine-N-[methoxy(polyethylene glycer-ol)-2000] micelles, or DSPE-PEG2000 and the mitochondrial GSH depleting agent diethyl maleate (DEM) for co-delivery in PDT. TPP-TK-PPa/DEM NPs have excellent mitochondrial targeting capabilities and can effectively deliver therapeutic agents to tumor cells and free ROS in mitochondria. At the same time, when administering PDT, TPP-TK-PPa/DEM NPs can increase ROS production in situ and simultaneously reduce ROS consumption by reducing intracellular GSH concentration under the action of slow release of DEM after cellular internalization. The increased release of ROS during PDT produces major changes in the morphology and physiology of the mitochondrial membrane with effects on accelerating cellular apoptosis phenomena. In vitro studies demonstrated that after PDT with TPP-TK-PPa/DEM NPs, remarkable cytotoxicity occurs in the MDA-MB-231 cell line, which is classified as “triple-negative” breast cancer. TPP-TK-PPa/DEM NPs provide a photosensitizer for PDT with a precise and efficient targeting capacity on tumor cells [59].

### 3.5. Future Perspectives on Mitochondrial Modulation in PDT

Small organic molecules excited by laser irradiation serve as fluorophores, widely used in medical imaging, fluorescence-guided surgery, and diagnostics. Research has recently focused on near-infrared (NIR) fluorophores (700–900 nm), forming the so-called in vivo optical window. Although many compounds have been proposed as PSs, the FDA has only approved four fluorophores for clinical trials: indocyanine green (ICG), methylene blue (MB), 5-aminolevulinic acid (ALA), and pafolacianine (OTL38) [60,61,62].

Bonelli et al. designed an innovative nano-PDT formulation consisting of mitochondria-targeted coumarin-PS mixed into amphoteric polyurethane-polyurea hybrid nanocapsules (NCs). The encapsulation of polyurethane-polyurea hybrid NCs with coumarin-based fluorophores (COUPY 1 and 2) using ECOSTRATAR technology resulted in enhanced photostability, lower dark cytotoxicity, and improved in vitro photoactivity. PDT with NC-COUPY-2 in human HeLa, buffalo green monkey kidney (BGM) cells, and HeLa multicellular tumor spheroids (MTCS) demonstrated strong tumor inhibition, high phototoxicity, mitochondrial damage, ROS production, and apoptosis induction [63].

The great progress made in the last decades in preclinical research on deciphering the role of mitochondria in cancer cells, and the rapid implementation of precision medicine in clinical pathology have led to an increase in the survival time of cancer patients. It has been observed that mitochondria permanently require oxygen for the metabolic oxidative phosphorylation (OXPHOS) process in order to obtain the energy necessary for cellular survival. Obstruction of the OXPHOS metabolic pathway will lead to disruption of the membrane potential, dysfunction of mitochondrial morphology and respiration, decreased ATP production and increased ROS [64].

### 3.6. Mitochondrial Complex III and ROS Release

Mitochondrial complex III (CIII, or cytochrome bc1 complex; ubiquinol cytochrome c reductase) on the electron transport chain (ETC) of oxidative phosphorylation is today considered the main point of ROS release in mitochondria and in blocking the upstream electron flow, with the reduction of ROS production in mitochondria, which oxidize the substrates in complex I. Complex III is composed of two centers, one called Qo disposed towards the intermembrane space and the other Qi placed on the inner membrane facing the mitochondrial matrix. Antimycin A is a secondary metabolite produced by Streptomyces bacteria and has a role as an inhibitor of cellular respiration, especially on oxidative phosphorylation. Antimycin A acts on complex III and inhibits the Qi center with consequences on the increase of superoxide production in the Qo center, thus directing oxidants away from the antioxidant defense of the matrix. Once interfered with by the inhibitor, complex III releases ROS into the cytoplasm that will degrade mitochondrial subcellular organelles, leading to cellular dysfunction. Complex III of the mitochondrial electron transport chain (mETC) has the fundamental mission of activating autophagy, unlike antimycin A, which can inhibit autophagy [65,66,67].

### 3.7. Atovaquone and Its Impact on Mitochondrial Respiration

Atovaquone, an FDA-approved antimalarial drug, is a specific inhibitor of the mitochondrial electron transport chain at complex III, depletes mitochondrial membrane potential, increases oxidative stress in vitro [68], decreases hypoxia in vivo [69], and, in addition, it can improve the antitumor response [70,71,72,73].

### 3.8. Biomimetic Nanoparticles in Cancer Treatment

Li et al. synthesized a biomimetic nanoparticle considered as an oxygen reservoir in which they included the mitochondrial photosensitizer IR780, atovaquone (ATO), an inhibitor of mitochondrial respiration, and co-loaded them into the red blood cell membrane (RBCm), coated with perfluorocarbon (PFC) with a liposome core, named RBCm@ATO-IR780-PFC liposomes. The authors experimentally used in vitro cell cultures from the human gastric cancer line AGS, and in vivo, mice with tumors induced from CT26 colorectal cancer cells. Biomimetic nanoparticles with ATO under the action of PDT effectively inhibited mitochondrial respiration and reduced endogenous oxygen consumption, and PFC delivered exogenous oxygen in a large amount. These oxygen modulators reversed hypoxia in vitro and in vivo and had prominent antitumor action on targeted mitochondria [74]. Fluorescence imaging-guided PDT is attracting widespread interest due to its unique advantages, including non-invasiveness, high spatiotemporal visibility, minimal side effects, and ease of use compared to conventional therapy procedures [75,76].

### 3.9. Photosensitizers and Aggregation-Induced Emission (AIE)

Traditional photosensitizers have rigid planar structures, are hydrophobic, and under physiological conditions tend to aggregate, which causes them to lose their fluorescence properties and consequently, their singlet oxygen-emitting power, a process called aggregation-induced quenching (ACQ). This phenomenon has led to research that has observed a bright emission from solid-state luminogenic dyes, a property known as AIEgen. The discovery of these properties of highly fluorescent AIEgens may be useful for monitoring and ameliorating the release of increased ROS and encouraging effects in suppressing cancerous lesions [77,78].

### 3.10. Design of Organic AIEgens for Cancer Therapy

Zhao et al. advanced a molecular design strategy to produce organic AIEgens to facilitate fluorescence imaging (FLI)-guided diagnosis and highly cost-effective photoinduced specificity in cancer therapy. The authors succeeded in designing four theranostic agents as aggregation-induced emission (AIE)-based photosensitizers (TPAPyTZ, TPAPyTC, TPAPyTM, and TPAPyTI) that have specific AIE properties. TPAPyTZ and TPAPyTC were attested for precise Golgi apparatus (GA) targeting, which could lead to cancer cell death. In vitro and in vivo studies have demonstrated that TPAPyTZ and TPAPyTC possess potent AIE properties, potent type I/II ROS scavenging capabilities, high photostability, precise GA-specific targeting, activation of mitochondrial apoptosis during PDT, and very good imaging quality. AIEgens of the TPAPyTZ and TPAPyTC-type have demonstrated their photodynamic effect in vivo through a percentage of 88%, and, respectively, 69% inhibition in HCT116 bearing-tumor mice. The results provide a promising method for applications of AIEgens for fluorescence imaging-guided PDT [79].

### 3.11. Mitochondrial Targeting in Cancer Therapy

It has been observed that PDT targeting mitochondria is much more effective in cancer therapy, as mitochondria are the fundamental cellular organelles for cellular respiration and the convergence points of various signals to transduction pathways [80].

### 3.12. Cytotoxicity of PDT and Targeting Mitochondria

Given the very short nanosecond (10–320 ns) lifetime and narrow diffusion beam (10–55 nm) of ROS, the cytotoxicity of PDT correlates with the area of cellular organelles targeted by PS [81].

Cationic nanocarriers based on triphenylphosphine and pyridinium are the most widely used in the synthesis of PSs for targeting mitochondria, but they still have problems related to rapid elimination, severe toxicity to the immune system, and the existence of the phospholipid bilayer surrounding mitochondria, constituting a barrier to effective action. Therefore, there is a pressing need to design nanocarriers for mitochondrial-targeted PDT that have cost-effective and living system-friendly structures, i.e., non-toxic and free of immunological events [82,83].

Designing DNA-based nanostructures for PS manufacture could overcome the drawbacks associated with mitochondrial-directed PDT. To progress in this field, it is necessary to identify DNA structures that are stable in biological environments and target tumor tissues. Research has shown that DNA nanostructures encoded with the poly-AS1411 aptamer for PS loading would provide durability to the DNA nanostructures and multiple guide probes, thereby improving the intelligent and sensitive recognition of tumor structures for targeted phototherapy [84,85].

### 3.13. DNA Nanotechnology for Mitochondrial-Targeted PDT

Chen et al. designed and developed a DNA nanoclew encoded with poly-AS1411 aptamer (multifunctional DNA nanoclew-AS-AMD) loaded with mitochondrial-selective cationic PS (APNO) and paramagnetic Mn^2+^ for MRI and PDT targeting mitochondria. The AS1411 aptamer in AS-AMD was designed to increase tumor targeting accuracy and cellular uptake. Paramagnetic Mn^2+^ released into the acidic TME enhances MRI performance in tumor tissue, and then precisely targets via PDT. Selectively released cationic APNO targeted the mitochondrial membrane and released ROS that induced in vitro apoptosis in 4T1 breast tumor cells. In vivo research in 4T1-tumor-bearing mice demonstrated a 91.3% decrease in tumor volume and extended survival rate in the AS-AMD group treated with PDT. AS-AMD demonstrated a very good tumor targeting response in the 4T1 tumor-bearing mouse model, substantially improved MRI operation and PDT efficiency. This aptamer-encoded DNA nanotechnology delivery system offers a novel mitochondrial-targeted PDT platform strategy that can be tailored for the therapy of various diseases by changing the sequence of the aptamer region in the DNA fragment. This research proposes a biocompatible and versatile DNA nanoplatform for the delivery of cationic PSs targeting mitochondria and the advancement of novel nanotheranostic agents [86].

### 3.14. Mitochondria-Targeted Photosensitizers in Cancer Therapy

Because mitochondria produce cellular energy and regulate cellular apoptosis, they have become attractive targets for PS targeting in PDT. As previously discussed, current PSs targeting mitochondria rely on the structure of cationic molecules that will be attracted to the negatively charged mitochondrial membrane. In 2015, Zhang et al. developed the photosensitizer IR700DX-6T that targeted a translocator protein (TSPO) on the mitochondrial membrane of TSPO-positive breast cancer cells (MDA-MB-231). IR700DX-6T induced apoptosis of TSPO-positive breast cancer cells in vitro and exponentially inhibited in vivo tumor growth in MDA-MB-231-bearing mice [87].

TSPO is a protein consisting of 169 amino acids rich in tryptophan, located on the outer membrane of mitochondria, which participates in metabolic and immune regulatory processes, being recently considered as a tumor biomarker, whose expression correlates with tumor aggressiveness and is used for diagnosis in molecular imaging studies and as an attractive therapeutic target in cancer [88,89].

### 3.15. Colorectal Cancer and PDT

Colorectal cancer is the second leading cause of death from all forms of cancer and ranks third in incidence worldwide. In 2020, there were over 1.9 million new cases of colorectal cancer, of which over 930,000 deaths occurred worldwide. The highest incidence was in Europe, Australia, and New Zealand, with the highest mortality rate in Eastern Europe. It is estimated that by 2040, there will be 3.2 million new cases of colorectal cancer per year with an increase in incidence of 63% and deaths by 73% per year [90,91].

Based on a previous study, Zhou et al. investigated the effects of IR700DX-6T-PDT on CT26 and HCT11 colorectal cancer (CRC) cells, in vitro and in vivo in the BALB/c mouse model with CT26 tumor. The authors demonstrated that IR700DX-6T-PDT potently inhibited CRC proliferation both in vitro and in vivo. At the same time, IR700DX-6T-PDT induced pyroptosis, p38 phosphorylation, and caspase-3-dependent cleavage of gasdermin E (GSDME). The use of decitabine, a methyltransferase inhibitor, reversed GSDME by demethylation and enhanced the effect of IR700DX-6T-PDT. The combination of decitabine, IR700DX-6T-PDT, and an anti-PD-1 antibody overcame the immunosuppressive environment and reduced tumor volume in vivo in experimental animals, thus offering a promising strategy for the therapy of “cold” tumors [92].

### 3.16. Challenges in PDT Efficacy

The efficacy of PDT in solid cancers is conditioned by two essential aspects: the first would be related to the reduced partial pressure of oxygen due to excessive tumor proliferation and liquefaction necrosis, and the second aspect is related to the diffusion and reduced lifetime of ROS molecules [93,94,95].

Li et al. invented a nanoplatform to target mitochondria and deliver oxygen to improve the efficiency of PDT in eliminating cancerous tumors. This nanoplatform comprises eccentric hollow mesoporous organic silica nanoparticles (EHMONs) equipped with triphenylphosphine (CTPP) molecules for mitochondrial targeting, the chlorin e6 PS and perfluorocarbons (PFCs) that can be loaded with oxygen. The EHMONs-Ce6-CTPP@PFC nanoplatform experimentally used on triple-negative breast cancer cells and in 4T1 tumor-bearing mouse models had good biocompatibility, high mitochondrial targeting capacity, and high destructive effects due to the high singlet oxygen content [96].

### 3.17. Overcoming Biological Barriers in PDT

In the case of deep tumors, upconversion nanoparticles (UCNPs) are used as nanocarriers for PSs as they increase the efficiency of PDT in these pathologies, but there are still many impediments to overcome biological barriers for effective mitochondrial targeting. In this regard, Liu et al. designed and manufactured photodynamic nanoplatforms targeting CD44 positive cells and mitochondria by self-assembly of conjugated hyaluronic acid-conjugated-methoxy poly(ethylene glycol)-diethylenetriamine-grafted-(chlorin e6-dihydrolipoic acid-(3-carboxypropyl)triphenylphosphine bromide) polymeric ligands (HA-c-mPEG-Deta-g-(Ce6-DHLA-TPP)) and NaErF_4_:Tm@NaYF_4_ core–shell UCNPs (referred to as CMPNs). Testing of these CMPNs showed good stability and drug loading capacity as well as enhanced competence in generating singlet oxygen (^1^O_2_) under the action of laser light based on the fluorescence resonance energy transfer (FRET) mechanism. In vitro experimental research on the human lung adenocarcinoma cell line A549 proved increased cytotoxicity and substantially reduced viability of CD44 positive cancer cells after using CMPNs, followed by the administration of NIR laser radiation. This research revealed the crucial role facilitated by CD44 positive cells in stimulating endocytosis and the capacity of UCNPs as nanocarriers for CD44 targeting and delivery of Ce6 PS. The study results open up prospects for the innovation of special nanoagents in PDT, which can overcome the current limitations of NIR laser irradiation for targeting mitochondria in deep cancers [97].

### 3.18. Immune Response in PDT

Immune checkpoint blockade (ICB) is a major opportunity for anti-neoplastic immunotherapy, and immunogenic cell death (ICD) is considered a promising pathway for tumor cell elimination. This process stimulates the adaptive immune response of T cells and the formation of long-term immunological memory. PDT can upregulate the programmed cell death 1 (PD-1)/PD-ligand 1(PD-L1) inhibitors, which will block the immunosuppressive signaling pathway and stimulate antitumor immune responses, leading to the killing of primary tumor tissue and blocking metastasis [98].

One marker of favorable response to immunotherapy is increased expression of PD-L1, which can be induced by hypoxia-inducible factor 1-alpha (HIF-1-alpha). HIF-1α plays an important role in the cellular response to systemic oxygen concentrations and the transcription of over 60 genes, including vascular endothelial growth factor (VEGF) and erythropoietin. HIF-1α, VEGF, and erythropoietin are involved in increased oxygen delivery, cell proliferation, and survival, as well as glucose and iron metabolism. HIF-1α expression can be regulated by PDT through oxidative stress, hypoxia, and ROS levels. It appears that HIF-1α and signaling pathways involved in hypoxia induce PD-L1 expression in the tumor when PDT acts. Tumor-associated M2 macrophages (TAMs) and suppressive tumor immune microenvironment (TIME) facilitate tumor growth and metastasis [99,100,101,102,103].

### 3.19. PROTAC Nanoplatforms for PDT

Proteolysis-targeting chimera (PROTAC) is designed to target proteins and induce antitumor effects. PROTAC has emerged as a promising acquisition for targeted therapy in various types of cancers, but the beneficial effect is limited by the low distribution and concentration in the tumor. PROTACs are molecules with bifunctional activity consisting of the target protein ligand, a linker, and an E3 ubiquitin-protein ligase that triggers the formation of chains of linked ubiquitin molecules to target the protein for degradation via the ubiquitin-proteasome pathway. PROTACs as heterobifunctional molecules have the primary purpose of inducing the proteasomal degradation, not inhibiting the target [104,105,106].

In this regard, Tong et al. have developed a multifunctional PROTAC-PDT nanoplatform (dBET6@CFMPD) to treat breast cancer and its metastasis. The nanoplatform dBET6@CFMPD is obtained via the self-assembly between dBET6 (small-molecule degrader) and the photosensitizer chlorin e6 (Ce6)-modified MMP-2 (matrix metalloproteinase-2) sensitive peptide (FFRFKGPLGLAGC)-PEG-DSPE conjugates. The multifunctional dBET6@CFMPD nanoplatform was used for mitochondrial targeting via PDT. The experiment was done using murine 4T1 breast cancer cells, 4T1-Luc cells, murine E0771 breast cancer cells, and E0771-Luc cells. A large amount of ROS was generated, strong cytotoxicity, and severe tumor cell death were observed in vitro. The antitumor and anti-metastatic effects were evaluated in vivo on 4T1 and E0771 subcutaneous models of female BALB/c mice indicating the great beneficial potential of dBET6@CFMPD for the treatment of breast cancer models and brain metastasis, by integrating PDT-PROTAC combination therapy and TIME remodeling. The anti-metastatic effect and its mechanism were investigated in vitro and showed an association between bromodomain-containing protein 4 (BRD4) inhibitor and Ce6 that strongly suppressed cell migration. The beneficial anti-metastatic effect of dBET6@CFMPD was supported by three factors: the first was the downregulation of epithelial-mesenchymal transition (EMT) induced by BRD4 degradation; the next was given by MMP-2 which set in motion the formation of nanofibers to limit tumor cell migration; and the last was due to the decrease in cell viability and motility induced by PDT. The authors demonstrated that the multifunctional PROTAC-PDT nanoplatform (dBET6@CFMPD) displayed in vitro and in vivo high biodistribution and retention and could trigger a strong antitumor effect on primary and metastatic tumors, transforming into a valuable nanomedicine with great future applications for combination cancer therapy [107].

### 3.20. Induction of Immunogenic Cell Death (ICD)

Induction of ICD is considered a promising method for cancer treatment because it triggers immune responses that ultimately lead to tumor cell death or necrosis. PDT is part of the category of innovative approaches in the fight to eradicate tumor formations by stimulating PSs that induce ROS release and initiation of ICD. The photoimmunotherapy response faces several impediments, such as reduced immunogenicity and efficacy due to insufficient ROS production, uneven distribution of the PS, and low oxygen levels in TME [108,109].

Duan et al. developed Rh-PTZ nanoparticles, i.e., a mitochondria-targeted type-I photosensitizer (Rh-PTZ) based on rhodanine, for PDT in a hypoxic environment. The compound Rh-PTZ was fabricated based on a typical D-A configuration with intramolecular charge transfer (ICT) properties. To improve biocompatibility and biostability, the authors encapsulated hydrophobic Rh-PTZ in F127 (polyethylene-polypropylene glycol) and obtained Rh-PTZ nanoparticles (Rh-PTZ NPs), which were then studied for mitochondrial targeting, ROS generation, and induction of ICD activation abilities under PDT. The researchers demonstrated that Rh-PTZ vigorously targets mitochondria, inducing the release of O_2_–• and OH, thus causing dramatic mitochondrial dysregulation. In vitro study was performed on 4T1 cell culture, where the level of immunofluorescence, ROS, cytotoxicity, intracellular ATP concentrations were monitored, and the antitumor activity was investigated in vivo on a 4T1 cancer model induced in female BALB/c mice. PDT with Rh-PTZ NPs amplified the ICD effect produced by mitochondrial stress with stimulation of the immune response, thus favoring the maturation of dendritic cells, improving the volume of infiltrated CD8+ immune T cells, and attenuating the immunosuppressive TME. The authors developed a novel phenothiazine-based type I PS with mitochondrial targeting of hypoxic tumors, which may provide new opportunities in the development of cancer PDT by improving cancer ICD through mitochondrial oxidative stress [110] (Table 2).

## 4. Final Remarks and Conclusions

PDT as a form of phototherapy involves light and photosensitizing chemicals, PSs, which in combination with molecular oxygen trigger the death of cancer cells through phototoxicity. This relatively new and promising method has two major impediments: the photosensitizers, which do not always effectively fulfill their intended role; and secondly, the TME, which, being hypoxic, dramatically limits the effects of this therapy. Given the special characteristics manifested by its non-invasive nature, spatiotemporal control, negligible side effects, and low risk of drug resistance, PDT has aroused the interest of clinicians and is already approved for the therapy of skin, breast, lung, esophageal, head, and neck cancers, etc., [111,112].

### 4.1. Recent Advancements in PDT: New Approaches

Production of high cytotoxic singlet oxygen, even though it has many applications, including PDT, is effective in the liquid phase but less efficacious in solids, for example, in nanomaterials. Samperi et al. assembled a mixture of supramolecular materials that contained a PS entrapped in highly organized nanofibers and proved by scientific experiments their remarkable ability to generate (^1^O_2_). By microscopy of super-resolution radial fluctuations (SRRF), the tetrakis(4-carboxyphenyl)porphyrin was studied after being embodied within the fibers of only a few nanometers of a bis-imidazolium gelator, a procedure capable of exactly determining the location of the chromophore. High-efficiency (^1^O_2_) was given rise from the hybrid nanofibers upon irradiation, compared to the dissolved porphyrin, resulting in a maximum productivity with a starting 14-fold superior value from fibers, as detected experimentally compared to porphyrin. This experiment opens new avenues for the easy preparation of similar, highly versatile hybrid supramolecular nanomaterials, much more efficient than currently dissolved PSs, performing multiple functions to generate highly efficient (^1^O_2_) in PDT, as well as in other laser medical applications [113].

### 4.2. Future Directions and Innovations in PDT

The invention of advanced photosensitizers, as shown in Table 1, which are easier to produce, have an extended absorption scale and biocompatibility, better selectivity, and enhanced efficiency, is a pressing need for improving PDT in deep tumor therapy.

In addition, recent research shows the possibility of preparing inventive organic shuttle-like nanoassemblies, i.e., nanoshuttles (NSs) with different shapes by isomeric photosensitizers, which exhibit clear crystalline features and ordered molecular packing, enhancing in vivo PDT with an 81% inhibition rate on tumor growth. The NSs have demonstrated excellent targeting and deep tumor penetration capacity, and fast cellular internalization with high ROS release. These NSs pave the way for the development of high-performance platforms for PDT applied in cancer therapy [114].

**Figure 5:**
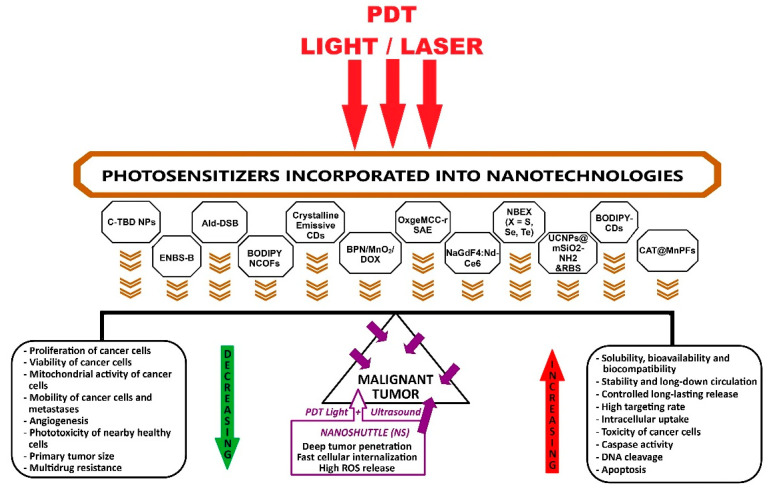
A complex representation of advanced applications of PSs incorporated in nanotechnologies for PDT. {Figure 1 was imagined and drawn by L.M.A. using Microsoft Paint 3D for Windows 10, being modified after Figure 4 [21]}.

An inspirational diagram of the state-of-the-art nano-photosensitizers, functional nanosystems, nanoscale delivery vehicles, and PDT-integrated nanoshuttles discussed in this review, which await successful application in clinical cancer therapy, is depicted in Figure 5.

### 4.3. The Role of Mitochondria in Cancer Cells and PDT

Great progress in the past decades in preclinical research on deciphering the role of mitochondria in cancer cells and the rapid implementation of precision medicine in clinical pathology have led to increased survival times for cancer patients. As shown in mammalian studies, mitochondria play a crucial role in cellular power supply by providing ATP from the tricarboxylic acid cycle (TCA) and oxidative phosphorylation. It has been observed that mitochondria constantly require oxygen for the metabolic process of phosphorylation to obtain the energy necessary for cellular survival. Obstruction of the OXPHOS metabolic pathway will lead to disruption of the membrane potential, dysfunction of mitochondrial morphology and respiration, decreased ATP production, and increased ROS [115,116].

### 4.4. Mechanisms of ROS Production and Its Impact on Cellular Health

Mitochondria are versatile and critical metabolic structures not only for energy production but also for modulating cellular immunity through several mechanisms, which generate cell apoptosis, ROS signaling, and DNA-dependent mitochondrial immune activation. These events are regulated by mitochondrial dynamics and mitochondrial damage-associated molecular patterns (mtDAMPs), complex phenomena by which mitochondria change, including their length and connectivity [117].

Production of ROS inside a cell can be achieved under the influence of several factors such as electron leakage during aerobic respiration, after inflammatory processes mediated by macrophages, under the action of ultraviolet rays, and many other external or stress stimuli. More and more publications have reported that mitochondrial ROS (mROS) plays a highly significant role in maintaining cellular, tissue, and whole organism homeostasis. mROS can modulate many signaling pathways and can stimulate the expressions of adaptation and defense of the organism in stress situations. mROS modulates fundamental physiological transformations related to cellular differentiation, proliferation, aging, and apoptosis. Increased ROS concentration, known as oxidative stress, will give rise to lipid peroxidation, protein carbonylation (the introduction of aldehyde or ketone carbonyl groups into the protein structure), DNA damage, and other changes, which will lead to pathologies such as senescence, metabolic, neurological, and other diseases, including cancer. Dysregulation of mitochondrial function will stimulate retrograde signaling pathways through specialized molecules in mitochondria such as ROS, calcium, oncometabolites, and exported mitochondrial DNA (mtDNA), as well as stress response pathways for the initiation, progression, and metastasis of cancer. Disruption of mitochondrial energy metabolism could eliminate the potential anticancer capacity of the immune system and amplify the phenomenon of cancer cell migration in the tumor microenvironment. All these recently proven aspects have inspired promising therapeutic strategies targeting mitochondria for the dissipation of malignant tumors through PDT [118,119].

### 4.5. PDT with Mitochondrial Targeting: A Promising Approach for Cancer Treatment

This is why PDT with specific photosensitizers targeting mitochondria is considered much more effective in cancer therapy, as mitochondria represent the fundamental cellular organelles for cellular respiration and the convergence points of various signals to cellular transduction pathways. PDT possesses a remarkable capacity in eliminating cancerous and non-oncological tumors through mechanisms of cell death induction, immune adjustment, etc. Since there is no protocol with specific criteria for each type of cancer for the use of a certain light source, a certain photosensitizer, and the appropriate dose, a percentage of patients do not respond satisfactorily to this treatment method and relapse or present metastases. On the other hand, in PDT, the fundamental pathophysiological mechanisms for cell death, which occur either through apoptosis, autophagy, or necrosis, are still not fully investigated. Currently, several references related to cell death pathways have appeared on this topic, such as necroptosis, mitotic catastrophe, paraptosis, and pyroptosis. A special type of cell death is ICD, which can also cause an immune response induced by antigens from destroyed cells, which in turn trigger very beneficial antitumor effects. The induced cell death pattern is conditioned by the laser or light dose, the subcellular site targeted by the PS, and the way in which it influences signaling pathways. Based on the above and the mechanisms of action, several treatment strategies are proposed by designing specifically targeted PSs and associating other therapeutic modalities such as nano-drugs, immunotherapy, and vaccines with the aim of inducing ICD, which would bring additional benefits to patients [116,120,121].

### 4.6. Challenges and Advances in PDT for Solid Tumors

One of the problems related to the efficiency of PDT applied in solid tumors is tumor hypoxia, aggravated especially by oxygen consumption during the therapeutic process, the rapid depletion of the optimal level of ROS due to high accumulations of GSH in the tumor microenvironment, and for these reasons, therefore, advancing these aspects is a permanent concern of researchers. Another impediment related to PDT is that most PSs put into practice have the inconvenience of being poorly soluble in water, with low specificity, and high toxicity in the dark.

Traditional PSs have inflexible planar structures, and, as mentioned, they are hydrophobic, and under physiological conditions, they tend to agglomerate with the loss of fluorescence properties and the ability to release singlet oxygen, a process known as aggregation-induced quenching. It is precisely this phenomenon that has prompted research that has observed that there is a bright emission of luminogenic dyes in the solid state, a property known as aggregation-induced emission (AIEgens). The discovery of these properties of highly fluorescent AIEgens may be useful for monitoring and ameliorating the release of an increased amount of ROS and encouraging effects in suppressing cancer lesions.

To improve the properties of PSs, there is currently great interest among researchers in finding nanocarriers such as inorganic nanoparticles (nonporous and mesoporous silica NPs, carbon-based NPs, quantum dots, and noble metal-based plasmonic NPs) and biodegradable organic NPs and their radiolabeled forms: liposomes, albumin-based nanoparticles, dendrimers, and polymeric nanoparticles, etc.

### 4.7. Future Directions: Mitochondrial-Targeted Nano-Photosensitizers

Mitochondrial-targeted nano-photosensitizers revealed in this review (see Table 2), designed with increased water solubility capabilities to extend the wavelength limits of deep tumor targeting by PDT, improve the specific delivery of PSs for certain tumor categories, and increase the efficiency of PDT in hypoxic TME, are highly promising, opening the way for coupling with other complex treatment procedures in molecular oncology.

This review should inspire researchers to contribute through creative approaches to optimizing PDT performance, imagining and designing new high-efficiency platforms for pharmaceutical formulations, smart PDT systems, and nanoshuttles for cutting-edge applications in molecular oncology and clinical cancer therapy.

### 4.8. Future Perspectives

Looking ahead, future advancements should focus on overcoming these limitations by developing novel PSs, including mitochondrial-targeted nano-photosensitizers. These new PSs, designed for increased solubility and targeted delivery, offer the potential for improved outcomes, particularly in challenging environments like hypoxic tumors. Additionally, combining PDT with other cancer therapies, such as chemotherapy, immunotherapy, and targeted molecular therapies, could provide synergistic effects that enhance therapeutic efficacy, reduce resistance, and improve patient survival.

The integration of PDT with combination therapies is a promising avenue for future research. By leveraging the strengths of each therapeutic modality, these approaches can help address the multifaceted nature of cancer, including tumor heterogeneity, resistance mechanisms, and the complex tumor microenvironment. For example, the use of PDT in conjunction with immunotherapies could stimulate immune responses while also directly killing cancer cells. Furthermore, nanotechnology offers the potential to design smart delivery systems that can selectively target tumors, reduce systemic toxicity, and maximize the effects of PDT.

Ultimately, continued innovation and interdisciplinary collaboration are essential to refine PDT protocols and develop novel combination strategies that will make PDT a cornerstone in modern cancer treatment regimens. With these efforts, PDT has the potential to become a first-choice treatment, providing patients with more effective, personalized, and less invasive options in the fight against cancer.

## Figures and Tables

**Table 1:** Experimental studies of nanotechnologies applied in the therapy of deep tumors through PDT.

Reference	Type of Study	Nanotechnology Tested	Results	Conclusions
Mao, D. et al., 2017, [24]	4T1 breast cancer cells were inoculated subcutaneously into normal BALB/c mice, resulting in a mammary tumor-bearing mouse model.	In vivo chemiluminescence of newly designed C-TBD NPs with ^1^O_2_ production.	C-TBD NPs showed high FR/NIR chemiluminescence and powerful singlet production in both primary and peritoneal metastatic mouse models. An innovative blueprint for simultaneous tumor diagnosis and treatment.	Selective chemiexcited image-guided and light-source-free PDT for deep tumors. Original platform for accurate cancer theranostics.
Li, M. et al., 2018, [25]	In vitro and in vivo models for PDT against hypoxic tumors. HepG2 or COS-7 cells, incubated on cell culture plates. Subcutaneous tumor model and in vivo cancer imaging in 6–8-week-old female Balb/c mice, subcutaneously injected with 1 × 10^6^ H22 cells to establish a liver tumor model.	Near-Infrared Light-Initiated Molecular Superoxide Radical Generator (ENBS-B with biotin ligand). ENBS-B or ENBS-C6-NH_2_ was injected intravenously into tumor bearing mice.	ENBS-B reaches 87- times higher cellular uptake in cancer cells than in normal ones. New options for personalized medicine and exceptional agents for clinical cancer therapy.	ENBS-B accurately targets neoplastic network cells and fully eliminates tumor growth upon low-dose light- irradiation.
Sun, CL. et al., 2019, [26]	In vitro Ald-DSB as 2PEF imaging on MCF-7 human breast cancer cells; and in vivo turn-on 2PE-PDT in a mice tumor model of melanoma. BALB/c nude mice were inoculated subcutaneously with B16-F10 melanoma cells.	In vitro 2PE-PDT on MCF-7 human breast cancer cells; laser irradiation at 760 nm (10 mW/cm^2^ 120 fs, 1000 Hz) for 20 min. For in vivo imaging and 2PA-PDT, mice were injected with Ace-DSB at the tumor location. Light treatment of 80 mW/cm^2^ was combined with Ace-DSB. 2PE-PDT was performed for 10 min, three times.	A simulation-assisted strategy for fast screening of small molecules to design turn-on-type 2PA dyes for simultaneous imaging and PDT of neoplasms. By turn-on only at tumor site, the phototoxicity to healthy cells can be decreased.	Practical implementation of the first screening for new theranostic chemical substances.
Guan, Q. et al., 2019, [27]	PDT in vitro and in vivo experiments on HeLa and MCF-7 cancer cell lines. Nude mice (BALB/c-nu♀, aged 5 weeks, 15~20 g) were subcutaneously injected MCF-7 cancer cells (5 × 10^6^ cells), to set up the MCF-7 xenograft model.	NCOF-based PSs for PDT: two BODIPY-decorated NCOFs and two amino-decorated BODIPY molecules. Green LED irradiation (40 mW/cm^2^), for different times: 0 (control) to 15 min.	PDT in vitro of nanoscale LZU-1-BODIPY-2I and LZU-1-BODIPY-2H, followed by high-efficacy PDT in vivo: successfully inhibited the MCF-7 xenograft growth without systemic toxicity, localized to lysosomes and mitochondria; cell death through mitochondrion-lysosome-connected pathways. Intracellular ^1^O_2_ imaging, cytostatic and other tests proved that both NCOF-based PSs are excellent nanomedical agents for PDT, especially the first one, with heavy atoms of iodine.	NCOF-based PSs tested for PDT proved to be high-performing agents, due to low cytotoxicity, fine biocompatibility, high uptake by cancer cells and highly efficient ^1^O_2_ generation. BDF procedure is yielding and many-sided for state-of-the art NCOF-based PDT materials, by covalently transfer of bioactive, chemotherapeutic, and targeting agents for clinical cancer treatment.
Wu, Q. et al., 2019, [28]	In vitro BPN/MnO_2_ -catalyzed ^1^O_2_ production within HeLa cells. In vivo efficacy against mice bearing HeLa tumors. In vivo antitumor effects of BPN/MnO_2_/DOX, followed by 660 nm photoirradiation and/or NIR 808 nm laser irradiation (0.8 W/cm^2^, 5 min).	Smart BPN/MnO_2_—tumor-driven nanotheranostic platform for T1-weighted MRI-guided synergistic PDT, PTT, and chemotherapy	Efficient ^1^O_2_ generation inside cancer cells under laser irradiation (660 nm, 0.22 W/cm^2^, 10 min). Significantly enhanced PDT and PTT performance. Outstanding antitumor performance of BPN/MnO_2_/DOX-based multimodality therapy.	Facile synthesis of compact BPN/MnO_2_ nanohybrid platform, with following advantages: -finite systemic cytotoxicity; -reduced therapeutic resistance; -precise spatial selectivity; -easy operation. Multifunctional BPN/MnO_2_/DOX inhibited tumor growth without relapses.
Wei, S.M. et al., 2020, [29]	In vitro PDT for CDs on tumor cell lines of human hepatoma Bel-7402, human lung adenocarcinoma A549, human cervical carcinoma HeLa, as well as normal epithelial cells of L929. In vivo PDT for CDs: intravenously administered tumor-bearing mice. Irradiation with laser (589 nm, 0.5 W/cm^2^).	Quantitative analysis of intracellular O_2′_**^−^** levels with different CDs. In vivo fluorescence imaging: maximum fluorescence on tumor tissue occurred 4 h after injection.	Large-scale karyopyknosis and necrosis in tumor tissues with CD and laser irradiation. No significant morphological changes or side effects in vital organs.	Extensive generation of luminescent CDs exhibiting an outstanding power to photogenerate O_2′_**^−^** with pronounced photocytotoxicity. Exploratory implementations of carbon-based materials in PDT and photocatalytic reactions.
Wang, D. et al., 2020, [30]	In vitro PDT efficacy and biocompatibility of OxgeMCC-r SAE against 4T1 tumor cells. In vivo PDT on 4T1 tumor- bearing mice, divided into 4 groups (control, free Ce6, MCC, and OxgeMCC-r). Laser irradiation (671 nm laser, 100 mWcm^−2^, 5 min) at 6 h post intravenous injection.	OxgeMCC-r SAE followed in vivo MRI-guided tumor PDT.	Remarkable accumulation of OxgeMCC-r inside the tumor 6 h after injection. Shrinkage of tumor on MRI 24 h after injection and PDT. Approx. total tumor elimination 3 days after PDT.	OxgeMCC-r SAE, a prospective theranostic nanoplatform for MRI-guided cancer therapy.
Zheng, B. et al., 2021, [31]	In vitro and in vivo PDT for treating deep-tumor models at low laser irradiation (660 nm and 808 nm) on CT26 cells, SKOV3 human ovarian cancer cells; and SKOV3-tumor bearing mice, intravenously injected with 200 mL of the nanoconjugates (10 mg/mL). In vivo tumor site fluorescence intensity and T1 -weighted MRI 24 h post-injection of NaGdF_4_:Nd-Ce6 nanoconjugates. Mice were treated with an 808 nm laser for 30 min, at 80 mW/cm^2^.	NaGdF_4_:Nd-Ce6 as a PDT agent.	Avoiding intersystem crossing by applying inorganic lanthanide nanocrystals, which act directly on the triplet excited states of PS. Greater penetration depth of 808 nm light in solid tumors. Much more apoptotic cells in histological sections from the NaGdF_4_:Nd-Ce6-FA+ 808 nm laser group.	A completely new design for lanthanide-triplet NIR photosensitization for increased ROS in PDT. Decreased energy losses. Much increased efficacy for deep cancer treatments at ultralow irradiation.
Yao, Q. et al., 2022, [32]	In vitro and in vivo PDT on breast cancer cells (MCF-7) and MCF-7-adr, i.e., resistant adriamycin breast cancer cells. Irradiated for 10 min (660 nm, 15 mW/cm^2^). In vivo PDT of multiple tumor models. Tumor-bearing mice were treated with light irradiation (50 mW/cm^2^ for 10 min)	NBEX (X = S, Se, Te), as PS with a type-III mechanism.	NBEX quickly changes to NP in a hydrophilic environment. Very good tumor elimination action for various tumors (subcutaneous, primary or glioma) and inhibition of metastases.	NBEX, as an oxygen-independent PS, immediately transfers light energy to the RNA of neoplastic cells and kills them with exceptional efficiency, also stimulating host immune responses to fight metastases.
Lu, B. et al., 2024, [37]	In vitro PDT towards NIH 3T3 cells, and tumor HeLa cells.	3 novel PSs: BODIPY-CD complexes tested for concentrations from 12.5 to 200 μg/mL.	Maximum emission peaks for alkynyl-BODIPY dye and BODIPY-CD complex at 783 nm and 874 nm, respectively. BODIPY-β-CD had a faster and higher ROS formation rate compared to the other two. HeLa cells viability decreased to 20% (at 100 μg/mL) with laser irradiation.	BODIPY-β-CD complex had best PDT activity against HeLa cells.
Li, L. et al., 2024, [44]	In vitro experiments on U87 -human glioblastoma cells and on U-CH1 -human chordoma cell line. In vivo murine model of brain tumor xenograft. 2.0 W NIR excitation power, 20 min of irradiation time.	NO-releasing platform for UCNPs@mSiO_2_-NH_2_&RBS	In vitro NO set free after NIR illumination of the platform restricted migration, reduced cells activity to 50% and triggered necrotic and apoptotic effects on neoplastic cell lines. In vivo study showed a significant halt in tumor growth in the treated group of mice, without adverse reactions.	Recently developed photocontrolled NO release platform by NIR photoexcitation of UCNPs@mSiO_2_-NH_2_&RBS may constitute an effective and safe therapy option for solid tumors.
Qiao, Y. et al., 2024, [50]	In vitro PDT on murine breast cancer 4T1 cells (4T1). Hemocompatibility studied in red blood cells.	Multifunctional CAT@MnPFs with nanometric size, i.e., ~114 nm.	CAT@MnPFs display a top ^1^O_2_ production. CAT@MnPFs killed 4T1 cells upon 650 nm irradiation (100 mW/cm^2^) for 10 min at a rate of 80.1% in the H_2_O_2_ group. High biosafety, negligible hemolysis at even 300 μg mL^−1^.	CAT@MnPFs, can give rise to O_2_ for pushing upward PDT.

**Table 2:** Photosensitizers targeting mitochondria in PDT.

References	Type of Study	Photosensitizer	Forms of Cancer	Effects on Mitochondria
Yang, Z. et al., 2019, [52]	In vitro and in vivo experiments	Mn_3_O_4_@MSNs@IR780 nanoparticles	Human gastric cancer cell line MKN-45P and tumor-bearing MKN-45P xenograft mice	Mn_3_O_4_@MSNs@IR780 can prevent tumor hypoxia in vivo. They offer highly targeted qualities towards mitochondria, with the ability to sustainably inhibit tumor hypoxia.
Wen, J. et al., 2021, [53]	In vitro and in vivo experiments	Multifunctional 3BP@PLGA-IR780 nanoplatform	Murine breast cancer line 4T1 cells and 4T1 tumor-bearing mice models	3BP@PLGA-IR780 nanoplatform penetrates deeply into the tumor, reduces tumor cells’ oxygen levels, enhances ROS generation, precisely targets and destroys tumor cell mitochondria.
Nash, G.T. et al., 2021, [54]	In vitro and in vivo experiments	New nanoscale metal–organic layer (nMOL) assembly, ZnOPPc@nMOL, with ZnOPPc [ZnOPPc = zinc(II)-2,3,9,10,16,17,23,24-octa(4-carboxyphenyl phthalocyanine] or ZnOPPc@nMOL	MC38 tumor-bearing C57BL/6 mice and CT26 tumor-bearing BALB/c mice	PDT with ZnOPPc@nMOL demonstrated mitochondrial penetration and >99% tumor growth inhibition efficiency, and 40–60% cure rates on 2 mouse models of colon cancer.
Cai, X. et al., 2021, [55]	In vitro and in vivo experiments	Multifunctional Ir-NPs nano-complex	SKOV3 ovarian carcinoma cell line and SKOV3 tumor-bearing mice	In vitro and in vivo Ir-NPs administered in PDT penetrated deeply into the tumor, disrupted mitochondrial redox homeostasis and induced apoptosis.
Huang, Z. et al., 2022, [59]	In vitro experiments	Amphiphilic heterodimeric photosensitizer (TPP-TK-PPa) and TPP-TK-PPa/DEM NPs nanoplatform	MDA-MB-231 breast cancer cell line as “triple-negative” breast cancer	TPP-TK-PPa/DEM NPs applied in PDT produced remarkable cytotoxicity in MDA-MB-231 cell line and may provide precise and efficient targeting capability on tumor cells.
Bonelli, J. et al., 2022, [63]	In vitro experiments	Coumarin PS in amphoteric polyurethane-polyurea hybrid nanocapsules. Nanoformulation NC-COUPY 2	Human cervix adenocarcinoma cell line. HeLa, buffalo green monkey kidney cells, BGM and MTCS	In vitro NC-COUPY 2 experimentally PDT demonstrated potent tumor growth inhibition effect, high phototoxic profile, mitochondrial degradation through autophagy, and apoptosis of cancer cells compared to free coumarin.
Li, X. et al., 2022, [74]	In vitro and in vivo experiments	RBCm@ATO-IR780-PFC liposomes	Human gastric cancer line AGS and in vivo, mice with tumors induced by CT26 colorectal cancer cells	Biomimetic nanoparticles with ATO under the action of PDT reversed hypoxia in vitro and in vivo and had prominent antitumor action on targeted mitochondria.
Zhao, X. et al., 2024, [79]	In vitro and in vivo study	Theranostic agents (TPAPyTZ, TPAPyTC, TPAPyTM, and TPAPyTI	HCT116 and HT29 colorectal cancer cells and HCT116-tumor bearing mice	TPAPyTZ induced GA-ROS, leading to GA breakdown, and activation of mitochondria caspase-related apoptosis, i.e., vigorous apoptosis of cancer cells.
Chen, M. et al., 2024, [86]	In vitro and in vivo study	Multifunctional DNA nanoclew-AS-AMD	Study on 4T1 breast tumor cells and 4T1-tumor- bearing mice	The intelligent poly aptamer-encoded DNA nanoclew activated mitochondria-targeted PDT and MRI. The AS-AMD platform substantially improved MRI performance and PDT efficacy.
Zhou, Y. et al., 2024, [92]	In vitro and in vivo study	IR700DX-6T-PDT	Human CRC cell lines, Murine colon adenocarcinoma cell line (CT26), HCT11 CRC cells, and BALB/c mouse model with CT26 tumor	IR700DX-6T-PDT induced pyroptosis in CRC in experimental mice via the ROS/p38/CASP3/GSDME axis and in this way could activate microsatellite stable colorectal cancer (MSS-CRC) to PD-1 blockade therapy.
Li, J. et al., 2024, [96]	In vitro and in vivo study	EHMONs-Ce6-CTPP@PFC nanoplatform	4T1 human breast cancer cells and 4T1 breast tumor model by BALB/c mice	EHMONs-CTPP-Ce6@PFC nanoplatform has good biocompatibility and enhanced efficiency in targeting mitochondria, alleviating hypoxia and generating singlet oxygen.
Liu, Z.H. et al., 2024, [97]	In vitro study	CMPNs photodynamic nanoplatform targeting CD44 and mitochondria	Human lung adenocarcinoma cell line A549	FRET mediation and mitochondria targeting with no cytotoxicity from CMPN-treated A549 cells. CD44 and mitochondria-targeted nanoplatform improved ^1^O_2_ production, mitochondrial targeting, mitochondrial depolarization and FRET-mediated PDT in CMPNs is a powerful master plan for enhanced PDT.
Tong, F. et al., 2024, [107]	In vitro and in vivo study	Multifunctional PROTAC-PDT nanoplatform (dBET6@CFMPD)	Murine 4T1 breast cancer cells, and 4T1-Luc cells Murine E0771 breast cancer cells and E0771-Luc cells. Brain-metastasis model of breast cancer 4 T1-Luc cells or E0771 Luc cells	dBET6@CFMPD platform targeting mitochondria in vitro and in vivo confirmed that it can stop breast cancer progression and its metastases through the association of PROTAC-PDT and TIME remodeling.
Duan, Z. et al., 2025, [110]	In vitro and in vivo study	Rh-PTZ NPs type I photosensitizer targeting mitochondria	4T1 cancer cell lines and 4T1 tumor carriers by female BALB/c mice	Rh-PTZ administered in PDT on 4T1 cancer cells lines and 4T1 tumor-bearing mice, confirmed mitochondrial targeting, ROS generation, ICD activation, apoptosis and necrosis of tumor cells.

## Data Availability

The data (selected literature) presented in this review article are available on request from the first author.

## Abbreviations

Ace-DSB	acetal-terminated distyrylbenzene derivative
ACQ	aggregation-induced quenching
AI	artificial intelligence
AIE	aggregation-induced emission
AIEgens	aggregation-induced emission luminogens
ALA	5-aminolevulinic acid
Ald-DSB	aldehyde-terminated molecules
AS-AMD	poly-AS1411 aptamer
ATO	atovaquone
ATP	adenosine triphosphate
BDF	bonding defects functionalization
BET	extra-terminal domain
BGM	buffalo green monkey kidney
BODIPY	boron-dipyrromethene
BODIPY-CDs complexes	BODIPY-α-CD, BODIPY-β-CD, and BODIPY-γ-CD where CD = cyclodextrin
3BP	3-bromopyruvate
BPN	black phosphorus nanosheets
BPN/MnO_2_	nanosponge from tightly packed MnO2-laden BPN
BPN/MnO2/DOX	BPN/MnO2 nanocomposite loaded with DOX
BRD4	bromodomain-containing protein 4
CAT@MnPFs	catalase-loaded manganese-porphyrin frameworks
CD	cyclodextrin
CDs	carbon dots
Ce6	chlorin e6
CIII	mitochondrial complex III; cytochrome bc1 complex; ubiquinol cytochrome c reductase;
CMPNs	self-assembly of hyaluronic acid-conjugated-methoxy poly(ethylene glycol)-diethylenetriamine-grafted-(chlorin e6-dihydrolipoic acid-(3-carboxypropyl)triphenylphosphine bromide) polymeric ligands (HA-c-mPEG-Deta-g-(Ce6-DHLA-TPP)) and NaErF_4_:Tm@NaYF_4_ core-shell UCNPs
COFs	covalent organic frameworks
COUPY 1	coumarin-based fluorophores 1
COUPY 2	coumarin-based fluorophores 2
CPPO	bis [2,4,5-trichloro-6-(pentyloxycar-bonyl)phenyl]oxalate
C-TBD NPs	C-TBD nanoparticles
CTPP	triphenylphosphine
dBET6	small-molecule degrader
DCs	dendritic cells
DEM	diethyl maleate
DNA	deoxyribonucleic acid
DOX	doxorubicin
EHMONs	eccentric hollow mesoporous organic silica NPs
EMT	epithelial-mesenchymal transition
ENBS-B	molecular superoxide radical (O_2_–•) generator
ETC	electro-transport chain
FDA	U.S. Food and Drug Administration
FEITC	β-phenylethyl isothiocyanate
FFRKGPLGLAGC-PEG-DSPE	DSPE-PEG_2000_-modified transformable peptides
FLI	fluorescence imaging
FRET	fluorescence resonance energy transfer
FR/NIR	far-red/near-infrared
GSDME	gasdermin E
GSH	glutathione
H_2_O_2_	hydrogen peroxide
HeLa	cervical cancer cell line containing human papillomavirus 18 (HPV-18)
HIF-1-alpha	hypoxia-inducible factor 1-alpha
HPV-18	human papillomavirus 18
ICB	immune checkpoint blockade
ICD	immunogenic cell death
ICG	indocyanine green
Ir	iridium
IR780	IR-780 dye for near-infrared fluorescence imaging/photosensitizer
MB	methylene blue
MC	Mn_3_ [Co(CN) 6]_2_
MC-r	Mn_3_ [Co(CN) 6]_2_ -Ru
MCF-7	human breast cancer cell line
MDA-MB-231	TSPO-positive breast cancer cells
mETC	mitochondrial electron transport chain
MKN-45 cells	human gastric adenocarcinoma cell line
MRI	magnetic resonance imaging
mROS	mitochondrial ROS
MSNs	mesoporous silica nanoparticles
Mn_3_O_4_@MSNs@IR780	NPs-PS (IR780)—90 nm MSNs—capping surface pores 5 nm Mn_3_O_4_ NPs
mtDAMPs	mitochondrial damage-associated molecular patterns
mtDNA	mitochondrial DNA
MTCS	HeLa multicellular tumor spheroids
NaGdF_4_:Nd	nanocrystals
NBEX (X = S, Se, Te)	type-III PS designed to bind to RNA
NCs	nanocapsules
NCOFs	boron-dipyrromethene (BODIPY)-decorated nanoscale COFs
NIR	near infrared
nMOL	nanoscale metal-organic layer
NO	nitric oxide
NP	nanoparticle
NPs	nanoparticles
NSs	nanoshuttles
^1^O_2_	singlet oxygen
OTL38	pafolacianine
OxgeMCC-r SAE	OxgeMCC-r single-atom enzyme (SAE)
OXPHOS	oxidative phosphorylation
2PA	two-photon absorption
PD-1	programmed cell death-1
PD-L1	programmed death ligand 1
PDT	photodynamic therapy
PFC	perfluorocarbon
PFCs	perfluorocarbons
PLGA	polylactic-co-glycolic acid
PpIX	Protoporphyrin IX
PROTAC	proteolysis-targeting chimeras
PTT	photothermal therapy
RBCm	red blood cell membrane
RBS	Roussin’s black salt
Rh-PTZ NPs	Rh-PTZ nanoparticles
ROS	reactive oxygen species
SAE	single-atom enzyme
SBU	secondary building units
SRRF	super-resolution radial fluctuations
TAMs	tumor-associated M2 macrophages
TBD	TPE-BT-DC
TCA	tricarboxylic acid cycle
TIME	tumor immune microenvironment
TME	tumor microenvironment
TNBC	triple-negative breast cancer
TPP-TK-PPa	amphiphilic heterodimeric photosensitizer
TSPO	translocator protein
UCNPs	upconversion nanoparticles
UCNPs@mSiO_2_-NH_2_&RBS	platform with nitric oxide release upon NIR irradiation
US	United States
VEGF	vascular endothelial growth factor
WHO	World Health Organization
ZnPc	zinc phthalocyanine
ΔΨm	mitochondrial membrane potential
Increased	↑
Decreased	↓
Present	+
Absent/Missing	-
